# Influenza-Associated Hospitalization in a Subtropical City

**DOI:** 10.1371/journal.pmed.0030121

**Published:** 2006-03-07

**Authors:** Chit Ming Wong, Lin Yang, King Pan Chan, Gabriel M Leung, Kwok H Chan, Yi Guan, Tai Hing Lam, Anthony Johnson Hedley, Joseph S. M Peiris

**Affiliations:** 1University of Hong Kong, Department of Community Medicine, Hong Kong, Hong Kong Special Administrative Region, China; **2**University of Hong Kong, Department of Microbiology, Queen Mary Hospital, Hong Kong, Hong Kong Special Administrative Region, China; Royal Free and University College Medical SchoolUnited Kingdom

## Abstract

**Background:**

The impact of influenza on morbidity and hospitalization in the tropics and subtropics is poorly quantified. Uniquely, the Hong Kong Special Administrative Region has computerized hospital discharge diagnoses on 95% of total bed days, allowing disease burden for a well-defined population to be accurately assessed.

**Methods and Findings:**

Influenza-associated morbidity and hospitalization was assessed by Poisson regression models for weekly counts of hospitalizations in Hong Kong during 1996 to 2000, using proportions of positive influenza types A (H1N1 and H3N2) and B isolations in specimens sent for laboratory diagnosis as measures of influenza virus circulation. We adjusted for annual trend, seasonality, temperature, and relative humidity, as well as respiratory syncytial virus circulation. We found that influenza was significantly associated with hospitalization for acute respiratory disease (International Classification of Diseases version 9 codes [ICD9] 460–466 and 480–487) and its subcategory pneumonia and influenza (ICD9 480–487) for all age groups. The annual rates of excess hospitalization per 100,000 population for acute respiratory diseases for the age groups 0–14, 15–39, 40–64, 65–74, and 75+ were 163.3 (95% confidence interval [CI], 135–190), 6.0 (95% CI, 2.7–8.9), 14.9 (95% CI, 10.7–18.8), 83.8 (95% CI, 61.2–104.2), and 266 (95% CI, 198.7–330.2), respectively. Influenza was also associated with hospitalization for cerebrovascular disease (ICD9 430–438) for those aged over 75 y (55.4; 95% CI, 23.1–87.8); ischemic heart disease (ICD9 410–414) for the age group 40–64 y (5.3; 95% CI, 0.5–9.5) and over 75 y (56.4; 95% CI, 21.1–93.4); and diabetes mellitus (ICD9 250) for all age groups older than 40 y.

**Conclusions:**

Influenza has a major impact on hospitalization due to cardio-respiratory diseases as well as on cerebrovascular disease, ischemic heart disease, and diabetes mellitus in the tropics and subtropics. Better utilization of influenza vaccine during annual epidemics in the tropics will enhance global vaccine production capacity and allow for better preparedness to meet the surge in demand that is inevitable in confronting a pandemic.

## Abstract 2

Influenza was found to have a major impact on hospitalization in Hong Kong due to respiratory diseases, cardiovascular disease, and diabetes. Previously, little was known about this impact in the tropics and subtropics.

## Introduction

Influenza is a major cause of acute respiratory diseases. Although the symptoms of influenza infection may be relatively mild and self-limiting, its complications can lead to hospitalization or death, especially in the older population or in those with chronic health problems [ 1].

Excess mortality and hospitalization associated with influenza have been used to measure influenza severity for over a century [ 2, 3, 4]. Two methods have previously been used to derive such estimates. One is a comparative method, in which the average numbers of deaths or hospital admissions during the months assumed to have low or no influenza virus circulation are defined, followed by calculation of the excess mortality or hospitalization by subtracting this baseline from the observed numbers of deaths or hospital admissions during influenza epidemics [ 5]. The other is the regression method developed by Serfling, which sets a baseline for excess numbers of events by fitting a linear regression function to the data of the period assumed to have low virus circulation, after taking into consideration the confounding factors such as seasonality and meteorological conditions [ 6]. The Serfling method was used to assess impact on hospitalization, but only in temperate countries where there are well-established and clear seasonal patterns of influenza [ 7, 8]. However, in tropical and subtropical regions such as Hong Kong, there are no well-defined seasonal patterns and the Serfling method cannot be readily applied. Using the comparative approach, excess hospitalization from pneumonia and influenza due to influenza A (H3N2) alone, and influenza A (H1N1) and influenza B together, from 1969 to 1995 in the United States was estimated to be 63.0 and 31.6 per 100,000 population, respectively [ 5]. In general, adults aged over 65 y and children younger than four y old are at higher risk of influenza-associated hospitalization. For children in the United Kingdom and the United States, influenza was found to be associated with excess hospitalization with annual rates of 41–186 per 100,000 population for cardiopulmonary diseases in the 1–14 age group [ 9], and 192–228 per 100,000 person-years for acute respiratory diseases in the 5–17 age group [ 10]; and was associated with a laboratory-confirmed hospitalization rate of 340–350 per 100,000 person-year in the less-than-2-y-old age group [ 11]. In Hong Kong, the influenza-associated excess hospitalization rates per 100,000 population for acute respiratory diseases were 279–288 in children younger than 1 y; 209–218 in children between 1 y and 2 y old; 77–126 between 2 y and 5 y old; 21–57 between 5 y and 10 y old; and 8–16 between 10 y and 15 y old [ 12]. In a community-based study, influenza was found to contribute substantially to health resource utilization [ 13]. In addition, influenza virus A (H3N2) has been demonstrated to result in more hospitalizations or deaths than influenza A (H1N1) and B [ 14]. Researchers in temperate regions in Europe and North America have used different strategies to quantify the disease burden associated with influenza [ 1, 15– 17].

However, application of either comparative or Serfling methods requires a well-defined seasonal pattern. In tropical and subtropical regions, influenza viruses circulate throughout the whole year without a clearly demarcated and predictable epidemic peak or period of virus activity. Therefore, assessment based solely on the peak epidemic periods will underestimate the disease burden of influenza [ 3]. As a consequence, there are few reports on influenza-related excess mortality or hospitalization in tropical and subtropical regions. Recently, Poisson regression was used to estimate influenza-related mortality in the United States [ 18] and by our group in Hong Kong [ 19]. Instead of using the observed numbers of deaths and excess deaths to define influenza epidemics, we took advantage of available virology surveillance data and calculated the excess mortality as the difference between the expected values when the observed proportion of virus isolations is fitted and the values when no virus circulation is assumed. In this approach the errors introduced by subjectively defining periods of high and low influenza virus activity can be minimized. This method allows estimation of disease burden even in the absence of clearly predictable and seasonal peaks of influenza activity [ 19]. This approach was recently used to estimate influenza-associated hospitalization in the United States [ 17] and in this study, in Hong Kong.

The Hong Kong Special Administrative Region (SAR) is situated in the tropics and has a subtropical climate with a mean temperature of 24° C and mean relative humidity of 79%. It has a well-defined population of 6.8 million, 95% of whom are Chinese, living in a compact geographical area. With most hospital admissions occurring in the public hospital sector, Hong Kong provides a unique opportunity to investigate influenza-associated hospitalization in a tropical or subtropical setting.

## Methods

### Data

**Figure 1:**
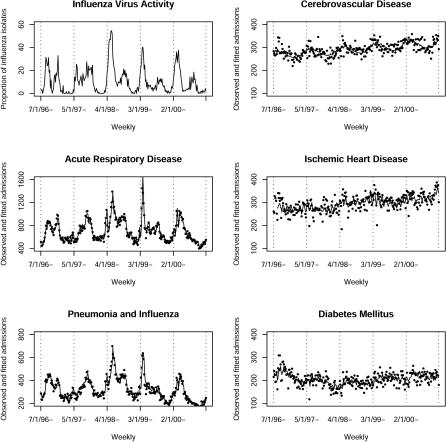
Weekly Proportion of Influenza A and B Isolates and Weekly Number of Hospitalizations for Five Causes Observed and Fitted According to Core Model with Control for Seasonality for All Ages

The public sector Hospital Authority manages over 95% of hospital bed days in Hong Kong, and a central computerized clinical management system captures discharges from all patients [ 20]. We obtained weekly numbers of hospital discharge diagnoses from the 14 acute hospitals during 1996 to 2000. The disease categories, retrieved from these records were coded in the International Classification of Diseases version 9 codes (ICD9). They included acute respiratory disease (ICD9 460–519) and subcategory pneumonia and influenza (ICD9 480–487) for age groups 0–14, 15–39, 40–64, 65–74, and 75+; cerebrovascular disease (ICD9 430–438), ischemic heart disease (ICD9 410–414), and diabetes mellitus (ICD9 250) for age groups 40–64, 65–74, and 75+. Urinary tract infection (ICD9 590–599) was also analyzed to serve as an unrelated control disease category. The Microbiology Laboratory of Queen Mary Hospital receives a mean of 6,249 (range 3,098 to 8,333) specimens for diagnosis of respiratory infections annually, and given the compact geographical area of the Hong Kong SAR, its data represent influenza virus activity within the area under investigation. The proportion of weekly total specimens positive for influenza A and B was 10.5% (range 0%–54.6%) ( Figure 1) and for respiratory syncytial virus (RSV) 8.8% (0%–31.5%). Weekly influenza and RSV activity was assessed from the proportion of specimens positive for influenza virus A and B (influenza A+B) and RSV. Weekly mean temperature and relative humidity data were obtained from the Hong Kong Observatory [ 21] .

### Poisson Regression

We applied Poisson regression [ 18] ( Protocol S1) to obtain the core model, with the weekly counts of hospitalization for each disease category as the dependent variable and co-variates including weekly temperature and relative humidity, as well as terms for trend, defined by serial week numbers (denoted by t), and seasonality, defined by pairs of sin (2 π t k/52) and cos (2 π t k/52) (where week t = 1, 2, … , 260, and k is the number of seasonal cycles per year to be determined by spectral analysis) [ 22] ( Table S1). Plots of partial autocorrelation for the residuals were examined for any discernible patterns. Autoregression terms up to 9 wk were added until all autocorrelations did not exceed the 95% confidence interval (CI) (around ± 0.1). The variables influenza A + B and RSV were entered into the core model to assess the impact of influenza circulation with adjustment for RSV. Delayed effects (lag effects) of influenza were assessed by entering the variable measured at 0–3 wk preceding the health outcome and selecting the one with the most significant effect.

### Excess Hospitalization

The difference between the total observed and the total expected hospitalizations when the proportion of influenza viral isolations was assumed to be zero, was divided by the total observed to yield the percentage of influenza-associated hospitalization, i.e., percentage of excess hospitalization attributable to influenza [ 23]. We then multiplied this percentage of excess hospitalization by total hospitalizations per year to obtain the “excess number” and divided it by the population to obtain a rate of excess hospitalization per unit of population to facilitate comparison with other published studies. Results are expressed as excess percentages for hospitalization and as rates per 100,000 populations for influenza-associated hospitalization. The statistical packages S-plus and Stata 8.2 (Stata Corporation, College Station, Texas, United States) were used for analysis.

## Results

### Age-Specific Hospitalizations

Overall hospitalization rates per 100,000 population for acute respiratory diseases and subcategory “influenza and pneumonia” showed a J-shaped pattern with the lowest rate in the 15–39 age group. For cerebrovascular disease, ischemic heart disease, and diabetes the rates increased with increasing age ( Table 1).

**Table 1:**
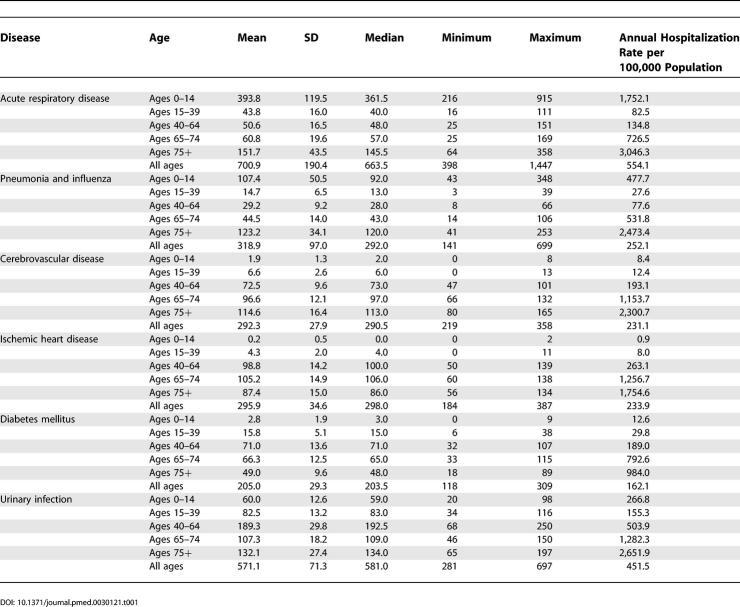
Weekly Number of Total Hospitalization and Annual Total Hospitalization Rate per 100,000 Population in Hong Kong (1996–2000)

### Poisson Regression Core Models

The observed weekly counts of hospitalizations, particularly for the two respiratory disease categories, closely followed the weekly counts of hospitalizations fitted from the Poisson regression core model and co-varied with positive influenza A and B proportion in specimens sent for laboratory diagnosis ( Figure 1).

### Influenza-Associated Excess Hospitalization

Acute respiratory disease and subcategory pneumonia and influenza were significantly associated with influenza ( *p* < 0.001) in all the age groups ( Table 2). For acute respiratory disease and for pneumonia and influenza, the influenza- associated excess hospitalization rates per 100,000 population were lowest (6.0 and 2.9, respectively) in the 15–39 age group, and highest (266.0 and 176.3, respectively) in the 75+ age group ( Table 2). Influenza was associated with 9.3% and 11.5% of total acute respiratory disease hospitalization in the 0–14 and 65- to 74-y age groups, respectively. Influenza was also significantly associated ( *p* < 0.001) with excess hospitalization for cerebrovascular disease and ischemic heart disease with rates of 55.4 and 56.4 per 100,000 population in those aged 75 y or older. In those 40–64 y of age, influenza was associated with 2.0% of all hospitalization for ischemic heart disease. In addition, influenza was significantly ( *p* < 0.01) associated with hospitalization for diabetes, with rates of 6.6, 23.9, and 53.3 per 100,000 population, respectively, in the age groups 40–64, 65–74, and 75+.

**Table 2:**
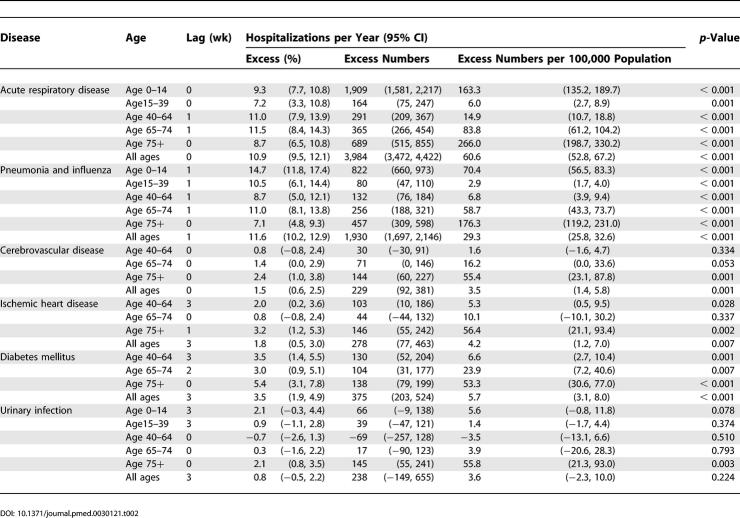
Influenza-Associated Excess Hospitalizations in 1996–2000 after Adjustment for Co-Variates Including Respiratory Syncytial Virus (1996–2000)

**Figure 2:**
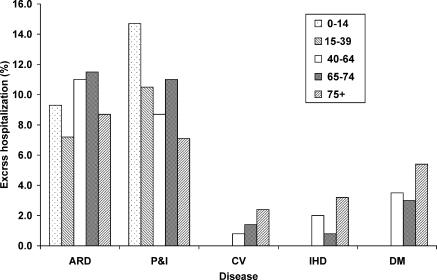
Percentage of Excess Hospitalizations Associated with Influenza at Different Ages ARD, acute respiratory diseases; P and I, pneumonia and influenza; CV, cerebrovascular disease; IHD, ischemic heart disease; DM, diabetes mellitus.

Considering all hospitalizations across all ages, influenza was associated with 10.9% (95% CI, 9.5–12.1) of total hospitalizations for acute respiratory diseases, 11.6% (95% CI, 10.2–12.9) for pneumonia and influenza, 1.5% (95% CI, 0.6–2.5) for cerebrovascular disease, 1.8% (95% CI, 0.5–3.0) for ischemic heart disease, and 3.5% (95% CI, 1.9–4.9) for diabetes ( Table 2, Figure 2). Influenza was not associated with hospitalization due to urinary tract infection for any of the age groups younger than 75, but there was an association in those 75 y and older.

## Discussion

Using the Poisson regression method we showed that influenza is strongly associated with cardio-respiratory hospitalization in all age groups in Hong Kong. We estimated that influenza led to 29.3 excess pneumonia and influenza hospitalizations per 100,000 populations of all ages, amounting to 11.6% of all hospitalizations for this disease category. A similar study in the United States during the period 1979 to 2001 found influenza associated with excess rates of pneumonia and influenza hospitalization of 36.8 per 100,000 populations for all ages annually, and accounted for 8.6% of all hospitalization for this diagnosis [ 17]. As expected, we demonstrate that those aged +75 y have the highest hospitalization rates and also the highest rate of influenza-associated hospitalizations in all disease categories studied. The association between influenza virus activity and hospitalization for acute respiratory diseases was least in those aged 15–39 y, a pattern also observed in the United States [ 17].

While the United States study [ 17] fitted monthly data with a single annual cycle, we chose the models that best fitted weekly data with changing patterns of seasonality year by year. A variable seasonal pattern is common in warm climates, a factor not usually allowed for in most other studies. McBean and Hebert [ 24] analyzed weekly hospital admissions of insurance beneficiaries in the United States and reported that influenza accounted for an extra 9.8% of pneumonia and influenza hospitalizations per year in the American population aged 65 y and over, compared to our estimate of 7.7% for Hong Kong ( Table S2). We simulated their analysis by changing their assumed seasonality of one cycle per year into two cycles per year since Hong Kong has two influenza peak seasons almost every year. Our results are close to their estimates, with an excess influenza-related hospitalization risk of 8.7% for pneumonia and influenza. We also applied their model to our datasets of hospital admissions for acute respiratory disease, cerebrovascular disease, ischemic heart disease, and diabetes and found all the excess hospitalization estimates to be similar to the estimates based on our model, except for diabetes (1.4% for McBean's versus 3.8% for our model) ( Table S2). In addition to the United States studies, one in the Netherlands [ 16] using Poisson regression on monthly data, showed significant excess hospitalization only for “pneumonia and influenza” with rates per 100,000 in the 65+ age group of 38–175 (in low and high risk groups), similar to those from this study and one United States study [ 17] of 97 and 37, respectively. Our model is better able to adjust for confounding due to irregular seasonal patterns, and differences between our estimates and those in the United States may also be explained by differences in the models used [ 12]. We have evaluated the fitness of core models to the original data through plotting partial autocorrelation function ( Figure S1) and thereby avoid the criticism leveled at previous studies about the lack of proof for model validity [ 25]. A further strength is that in our estimation for the excess hospitalization rates, we chose the most significant effect (i.e., with smallest *p*-value) within a lag period of 0–3 wk. This method allows us to estimate the effect for infection that may occur one to several weeks after exposure to viruses, or from admissions which may be delayed one to several weeks after infection. Selection of the most significant effect estimate during a lag period of several days or weeks is widely used in health effects of air pollution studies [ 26].

In addition to the marked increase in hospitalizations due to pneumonia and influenza, which would be expected to show an association with influenza virus activity, our results also show that influenza is associated with excess hospital admissions for other chronic diseases such as cerebrovascular disease, ischemic heart disease, and diabetes. Effects of influenza on cerebrovascular and ischemic heart disease may be partly explained by the acute pathophysiological responses triggered by influenza, which include changes in circulating clotting factors, platelet aggregation, and lysis, concentrations of inflammatory-response proteins, and alternations in cytokine concentrations [ 27, 28], which may enhance thrombotic tendencies, impair vasodilation, or even lead to endothelial injury [ 29]. Acute respiratory tract infection is reportedly associated with an increased risk of myocardial infarction and stroke [ 30]. We find that excess influenza-related hospitalizations, both for cerebrovascular disease and for ischemic heart disease, were statistically significant in those older than 65 y ( Table S2).

Hospitalization for diabetes mellitus was associated with influenza in those older than 40 y ( Table 2). Impaired immune responses in diabetic patients might predispose to increased direct and indirect effects of influenza, including bacterial secondary infection, and thereby lead to increased influenza-related morbidity and mortality [ 31]. Also, influenza and its complications may destabilize the health and glycemic control of chronically ill patients with diabetes so that they may be hospitalized for a range of diabetes-related complications that may or may not be overtly related to influenza. Our diabetic inpatients also had a higher case fatality ratio during influenza epidemic periods than the baseline (4.1% versus 3.3%, *p* value < 0.001) (unpublished data). So it is likely that protection against influenza could contribute to the reduction of both hospitalization and mortality in patients with diabetes [ 32].

Overall, influenza was specifically related to the excess risk of hospitalization with cardio-respiratory conditions, but not with urinary tract infection, which served as a control disease in this analysis. This suggests that the elderly who are a higher risk group for cardio-respiratory disease would benefit from an influenza prevention program. However, in the 75+ age group, influenza was significantly associated with a discharge diagnosis of urinary tract infection. This may be because clinical presentation of infections in the elderly is often atypical and non-specific, resulting in an imprecise final clinical diagnosis. Furthermore, older persons are more likely to be admitted with multiple diseases. The probability that patients with principal diagnosis of urinary tract infection had co-morbidity coded as pneumonia and influenza, cerebrovascular disease, ischemic heart disease, and diabetes mellitus increased with age and was 0.76%, 8.69%, 7.32%, and 7.20%, respectively, for 75+ comparing with 0.27%, 3.79%, 3.42%, and 6.70%, respectively, for the 65–74 age group (unpublished data).

The lack of convincing data of the clinical disease burden of influenza in warm climate countries has led to the under-utilization of influenza vaccine in the tropics [ 33]. For example, while Hong Kong has a higher per capita gross domestic product than Australia or New Zealand, the influenza vaccine utilization in these three regions and countries is respectively 28, 183, and 171 per 1,000 population in the year 2000, respectively [ 33]. The low vaccine uptake may also reflect inadequate reimbursement of the vaccine costs, the need for yearly vaccination, a perceived low efficacy, and fear of the relatively frequent, albeit mild, side-effects [ 34]. A previous study in children using a comparative approach demonstrated that influenza-associated hospitalization rates in Hong Kong were comparable to those in the United States [ 12]. In another recent study in Hong Kong, the seasons were arbitrarily defined and adjustment was based on models that did not recognize that count data should follow a Poisson distribution [ 35].

Our analysis using Poisson regression modeling of weekly data demonstrates an approach for assessment of disease impact of influenza in tropical and subtropical regions and shows that it is possible to estimate influenza disease burden in tropical regions with varying seasonal patterns of virus activity. The Poisson regression modeling method provides estimates that are robust to potential confounding effects, which may arise from uncontrolled personal factors, such as smoking status, and chronic co-morbidity [ 26]. This methodology correlates influenza virus activity with the residual variation remaining after subtracting the underlying seasonal variation in disease morbidity. However, some of the seasonal variation in disease morbidity may also be contributed by influenza, and this component is not captured by this type of analysis. Thus, the estimates derived from our analysis is a conservative one, and very likely to underestimate the true impact of influenza on hospitalization. Ideally, an approach that can also capture the seasonal variation associated with influenza virus circulation is needed.

Our results provide evidence for the identification of high risk groups in tropical and subtropical settings who would most benefit from influenza vaccination. This is also the first study showing that circulation of influenza viruses is related to hospitalization for diabetes mellitus supporting the argument that influenza vaccination may prevent hospitalization for persons with this chronic problem. Increased morbidity and mortality for diabetes during influenza epidemics have long been recognized [ 36], but we assessed the excess hospitalization rates due to yearly influenza virus circulation rather than epidemics alone.

Although a recent study has cast doubt on the extent of protective efficacy of vaccination in the elderly [ 7], those findings do not detract from the overall consensus that vaccination remains strongly recommended for protecting the elderly and others at high risk from the adverse effects of influenza [ 37]. Our findings emphasize that the disease burden associated with influenza encompasses not just acute respiratory diseases, but also cerebrovascular disease, ischemic heart disease, and diabetes mellitus. Vaccination for influenza is reported to result in a reduction in hospitalization for respiratory or chronic heart disease greater for those aged 65–79, than for those older than 80 [ 38], and also for cardiac disease, cerebrovascular disease, and for pneumonia and influenza [ 29] in the 65+ age group. According to a meta-analysis [ 39], the pooled estimate of vaccine efficacy in persons aged 65 y and older, in terms of relative risk reduction, was 0.27 (95% CI, 0.21–0.33) for preventing hospitalizations for pneumonia and influenza, 0.22 (95% CI, 0.15–0.28) for respiratory diseases, and 0.24 (95% CI, 0.18–0.30) for cardiac diseases. In this age group, the overall hospitalization rates per 1,000 populations were 12 for pneumonia and influenza, 68 for respiratory diseases, and 65 for cardiovascular diseases (derived from data in Table 1). Assuming that these hospitalization rates can approximate the expected hospitalization rates in the unvaccinated population, the number needed to treat [ 40, 41] to prevent a hospitalization was estimated to be 311 (95% CI, 255–401) for pneumonia and influenza, 71 (95% CI, 55–105) for respiratory diseases, and 68 (95% CI, 54–91) for cardiovascular diseases. However, these estimates of the number needed to treat tend to be conservative, since we use the hospitalization rates of the general population that are likely lower than those of unvaccinated groups. A further cost-benefit analysis based on our data may provide an assessment of the social costs of this disease in Hong Kong and the total value of the benefits that may accrue from a vaccination program.

Hong Kong may not be representative of all tropical regions in the demographics of health and sickness, in patterns of morbidity and mortality, and in socio-economic terms. However, it is pertinent to note that the increasing urbanization and the growth of mega-cities is a rapidly increasing global trend and these developments are occurring in tropical regions as well. As such, the experience of Hong Kong is relevant to the tropics and subtropics in general. Better utilization of influenza vaccine during annual epidemics will enhance local, regional, and global vaccine production capacity and also infrastructure for vaccine delivery [ 42]. Therefore, in addition to a direct benefit reduction of influenza-associated hospitalization, the greater utilization of influenza vaccines during inter-pandemic periods in the tropics will lead to better access to vaccine supplies when confronting a future pandemic.

## Supporting Information

Figure S1 Plots of Partial Autocorrelation for Residuals of Core Models Fitted to All Age Groups after Adjustment of Seasonality Partial ACF, partial autocorrelation function.
(3.8 MB JPG).

Protocol S1 Supplementary Notes for Statistical Methods (46 KB DOC).

Table S1 Numbers of Seasonal Periods (k) per Year Determined by Spectral Analysis (59 KB DOC).

Table S2 Estimated Percentages of Excess Hospitalizations Associated with Influenza in Hong Kong Using Our Model Compared with Those Using McBean's Model Long time trend, temperature, humidity, and RSV circulation are adjusted for in both McBean's and our model. In McBean's model, seasonality is adjusted by simply adding one pair (assuming one cycle of virus circulation per year) or two pairs of sinusoidal terms (assuming two cycles per year). Instead, our model uses spectral analysis to define appropriate cycles of virus circulation.
(22 KB DOC).

**Patient Summary**

**Background**

Influenza is a very common illness. It can sometimes be serious—for example, in the elderly—and it adds to the costs of health care. Studies in developed countries have shown that both death rates and the number of people admitted to the hospital rise during seasonal influenza outbreaks. These studies have made it possible to calculate the “disease burden” of influenza. So far, however, little has been known about the impact influenza has on people in tropical and subtropical countries. It is a difficult issue to study, partly because there is usually no clearly established seasonal pattern of influenza in these countries.

**Why Was This Study Done?**

The Hong Kong Special Administrative Region in China (population 6.8 million) has a subtropical climate. Ninety-five percent of the people admitted to the hospital go to public-sector hospitals, which have a central computerised system where clinical records are kept on all patients. This makes Hong Kong an excellent place to study the impact influenza has on a subtropical city.

**What Did the Researchers Do and Find?**

The researchers obtained information on patients admitted to hospitals in Hong Kong during 1996–2000. In order to analyze the information, they developed an appropriate statistical technique that allowed for such factors as variations in temperature and humidity. They found that during influenza outbreaks hospital admissions increased, not only for respiratory diseases (including pneumonia), but also for heart problems, stroke, and diabetes. The increases were most noticeable for older people. Overall, influenza was responsible for 10.9% of admissions for acute respiratory diseases, 1.5% of admissions for stroke, 1.8% for heart attacks, and 3.5% for diabetes. These figures are comparable to what has been found in developed countries outside the tropics.

**What Does This Mean?**

On the basis of this study, influenza seems to have a bigger impact on subtropical and tropical countries than has been recognized until now. Vaccination programs for people at high risk, mainly the elderly, could have many benefits. However, it should be noted that Hong Kong is not typical of the rest of the tropics and subtropics. Many tropical countries face a disease burden from other medical conditions that is much larger. However, a true understanding of the disease burden of influenza in relation to other medical conditions is important in order to make rational assessments of resource allocations in health care.

**Where Can I Find More Information Online?**

Fact sheets are available about various aspects of influenza from the Web site of the World Health Organization, which takes a global overview of the impact of the disease:

http://www.who.int/topics/influenza/en

There are many health Web sites aimed at patients that provide basic information about influenza. Some examples are:

http://www.niaid.nih.gov/publications/cold/sick.pdf

http://www.niaid.nih.gov/factsheets/flu.htm

http://www.cdc.gov/flu

http://jama.ama-assn.org/cgi/content/full/293/8/1024

http://www.bbc.co.uk/health/conditions/flu1.shtml

The Government of the Hong Kong Special Administrative Region has a Web site that includes facts and figures about health in Hong Kong:

http://www.healthyhk.gov.hk/phisweb/en

## Abbreviations

CI: confidence interval
ICD9: International Classification of Diseases version 9 codes
RSV: respiratory syncytial virus
