# Supplementary material for: Influenza-Associated Hospitalization in a Subtropical City
Source: PLoS Med. 2006 Mar 7;3(4):e121. doi: 10.1371/journal.pmed.0030121 (PMC1391978; doi:10.1371/journal.pmed.0030121)
Supplement: Supplementary file 1 [file pmed.0030121.sd001.doc]

###### Box S1: Supplementary notes for statistical methods

(a)	*Poisson regression*

We applied Poisson regression with weekly counts of hospitalization for each disease category as the dependent variable and weekly average of temperature and relative humidity as well as trend and seasonality as independent variables to fit the core model as

$$\begin{matrix} \log E(Y_{t})=\beta_{0}+\beta_{1}t+\sum_{i} \left(\beta_{2i}\sin(\frac{2t\pi k_{i}}{52})+\beta_{3i}\cos(\frac{2t\pi k_{i}}{52})\right)+\beta_{4}temp_{t}+\beta_{5}humid_{t} \\ +\beta_{6}RSV_{t}+\mu_{t} \end{matrix}$$

… [Equation 1]

where $t$ stands for serial week number from 1 to 260; $E(Y_{t})$ is the expected numbers of health outcome; $\beta_{0}$ is the intercept in the model; $\sin(\frac{2t\pi k_{i}}{52})$ and $\cos(\frac{2t\pi k_{i}}{52})$stands for the seasonality in which $k_{i}$ is the number of seasonal periods per year to be determined by spectral analysis; $temp_{t}$ for weekly average of temperature; $humid_{t}$ for weekly average of relative humidity and $RSV_{t}$ for weekly proportion of RSV isolation. If the plots of partial autocorrelation for residuals have any discernible patterns, then we add$\mu_{t}$, which are the autoregression terms up to 9 weeks, to the core model to eliminate those patterns.

After the core model has been obtained, we add the main effect of influenza, which is defined as the weekly proportion of influenza A+B, into the core model:

$$\begin{matrix} \log E(Y_{t})=\beta_{0}+\beta_{1}t+\sum_{i} \left(\beta_{2i}\sin(\frac{2t\pi k_{i}}{52})+\beta_{3i}\cos(\frac{2t\pi k_{i}}{52})\right)+\beta_{4}temp_{t}+\beta_{5}humid_{t} \\ +\beta_{6}RSV_{t}+\mu_{t}+\beta_{7}Flu_{t} \end{matrix}$$

… [Equation 2]

where $Flu_{t}$ is the weekly proportion of influenza and $\beta_{7}$ is the parameters for

weekly proportion of influenza A+B isolation.

*(b)	excess (%) and excess numbers per 100,000 population for elimination of weekly influenza proportion to zero:*

We defined the excess hospitalization number as the difference between the total observed and total expected hospitalization when the proportion of influenza viral isolations was assumed to be zero. We then divided the excess number by the total observed to obtain the excess (%) as below:

$excess(\%)=\frac{\sum_{t=1}^{260} (Y_{t}-E(Y_{t}|Flu_{t}=0))}{\sum_{t=1}^{260} Y_{t}}\times100\%$

where $E(Y_{t}|Flu_{t}=0)$ are weekly expected hospitalization under the assumption that the proportion of influenza viral isolations is zero.

We then multiplied the percentage excess by total hospitalization per year and divided it by the population to obtain a rate of excess hospitalization per unit of population:

$$\begin{matrix} excess numbers per 100,000 population \\ \text{ }=\text{ }\frac{excess(\%)\times average hospitalization\text{ }per\text{ }year}{average population\text{ }per\text{ }year}\text{ }\times\text{100,000} \end{matrix}$$

.
