# Supplementary material for: Influenza-Associated Hospitalization in a Subtropical City
Source: PLoS Med. 2006 Mar 7;3(4):e121. doi: 10.1371/journal.pmed.0030121 (PMC1391978; doi:10.1371/journal.pmed.0030121)
Supplement: Supplementary file 2 [file pmed.0030121.st001.doc]

| **Diseases** |  | | | | | | |
| --- | --- | --- | --- | --- | --- | --- | --- |
| **Acute Respiratory Disease** | **k** | | | | | | |
| age 0-14 | 0.2 | 0.6 | 1.0 | 1.8 |  |  |  |
| age15-39 | 0.2 | 0.4 | 1.0 | 1.8 | 2.0 |  |  |
| age 40-64 | 0.2 | 0.8 | 1.0 | 1.8 | 2.0 |  |  |
| age 65-74 | 0.2 | 0.8 | 1.0 | 1.8 | 2.0 |  |  |
| age 75+ | 0.2 | 0.8 | 1.0 | 1.8 | 2.0 |  |  |
| all ages | 0.2 | 0.6 | 1.0 | 1.8 |  |  |  |
| age 65+ | 0.2 | 0.8 | 1.0 | 1.8 | 2.0 |  |  |
| **Pneumonia and Influenza** |  |  |  |  |  |  |  |
| age 0-14 | 0.2 | 0.4 | 0.6 | 1.0 | 1.8 | 2.0 |  |
| age15-39 | 0.2 | 0.4 | 1.0 | 3.8 |  |  |  |
| age 40-64 | 0.2 | 0.8 | 1.0 | 1.8 | 2.0 |  |  |
| age 65-74 | 0.2 | 0.8 | 1.0 | 2.0 |  |  |  |
| age 75+ | 0.2 | 0.8 | 1.0 | 1.8 | 2.0 |  |  |
| all ages | 0.2 | 1.0 | 1.8 |  |  |  |  |
| age 65+ | 0.2 | 0.8 | 1.0 | 1.8 | 2.0 |  |  |
| **Cerebrovascular Disease** |  |  |  |  |  |  |  |
| age 40-64 | 24.0 | 24.8 |  |  |  |  |  |
| age 65-74 | 1.0 | 9.8 | 21.2 |  |  |  |  |
| age 75+ | 1.0 |  |  |  |  |  |  |
| all ages | 1.0 |  |  |  |  |  |  |
| age 65+ | 1.0 |  |  |  |  |  |  |
| **Ischemic Heart Disease** |  |  |  |  |  |  |  |
| age 40-64 | 0.4 | 1.0 | 8.0 |  |  |  |  |
| age 65-74 | 1.0 | 5.8 | 8.0 | 8.4 | 9.4 |  |  |
| age 75+ | 1.0 | 2.0 |  |  |  |  |  |
| all ages | 0.4 | 1.0 | 8.0 | 8.4 | 9.4 |  |  |
| age 65+ | 1.0 |  |  |  |  |  |  |
| **Diabetes Mellitus** |  |  |  |  |  |  |  |
| age 40-64 | 0.2 | 0.8 | 1.0 |  |  |  |  |
| age 65-74 | 0.2 | 0.4 | 1.0 | 6.0 |  |  |  |
| age 75+ | 0.2 | 1.0 | 8.0 |  |  |  |  |
| all ages | 0.2 | 1.0 |  |  |  |  |  |
| age 65+ | 0.2 | 0.4 | 1.0 |  |  |  |  |
| **Urinary Infection** |  |  |  |  |  |  |  |
| age 0-14 | 0.2 | 0.4 | 0.8 | 1.0 | 3.0 | 3.6 | 20.2 |
| age15-39 | 1.0 | 6.0 | 8.0 | 17.6 | 22.6 |  |  |
| age 40-64 | 0.2 | 0.4 | 1.0 | 1.2 | 4.0 |  |  |
| age 65-74 | 0.2 | 1.0 |  |  |  |  |  |
| age 75+ | 0.2 | 1.0 |  |  |  |  |  |
| all ages | 0.2 | 1.0 |  |  |  |  |  |
| age 65+ | 0.2 | 1.0 |  |  |  |  |  |
