# Supplementary material for: Influenza-Associated Hospitalization in a Subtropical City
Source: PLoS Med. 2006 Mar 7;3(4):e121. doi: 10.1371/journal.pmed.0030121 (PMC1391978; doi:10.1371/journal.pmed.0030121)
Supplement: Supplementary file 3 [file pmed.0030121.st002.doc]

| **Excess (%)**  **(95% CI)** | | **ischemic heart disease** | **Acute respiratory disease** | **P&I** | **Cerebrovascular** | **Diabetes mellitus** |
| --- | --- | --- | --- | --- | --- | --- |
| **McBean’s Model** | one cycle,65+ | 2.4  (1.1,3.8) | 13.5  (11.3,15.7) | 11.3  (9.2,13.4) | 2.0  (1.0,3.0) | 2.0  (0.2,3.9) |
|  | two cycles,65+ | 2.1  (0.4,3.8) | 10.5  (7.7,13.3) | 8.7  (6.1,11.2) | 2.0  (0.8,3.3) | 1.4  (-0.8,3.8) |
| **Our model** | 65+ | 2.3  (1.0,3.7) | 9.4  (7.4,11.4) | 7.7  (5.6,9.7) | 1.9  (0.8,2.9) | 3.8  (2.0,5.6) |
